# Opinions and evaluations of mathematics teachers on concrete models of their design in the context of positive psychology

**DOI:** 10.3389/fpsyg.2022.964991

**Published:** 2022-08-12

**Authors:** Meryem Gülyaz Cumhur, Murat Tezer, Tolgay Karanfiller, Ezgi Pelin Yıldız

**Affiliations:** ^1^Faculty of Arts and Sciences, Near East University, Nicosia, Cyprus; ^2^School of Applied Sciences, Cyprus International University, Nicosia, Cyprus; ^3^Department of Computer Technology, School of Technical Science, Kafkas University, Kars, Turkey

**Keywords:** pre-service teacher, primary mathematics education, positive psychology, concrete models, teaching mathematics

## Abstract

The purpose of this study is to investigate the opinions and evaluations of pre-service mathematics and pre-service primary school teachers regarding the concrete models of their design during the COVID-19 Pandemic in the context of positive psychology. In this study, a mixed research method, in which quantitative and qualitative research methods are used together was used. The participant group of the study consists of 441 pre-service mathematics and pre-service primary school teachers studying at universities. For the conduct of the qualitative research, 44 pre-service teachers were selected using the purposive sampling method. “Mathematics Teaching Practices Enriched with Concrete Models Perception Scale” developed by the researchers was used to collect the quantitative data to reveal the opinions of teachers regarding mathematics teaching practices enriched with concrete models. In this study, open-ended questions were used to obtain qualitative data, and the data were evaluated with content analysis. According to the results of the study, it was determined that the majority of the pre-service teachers had positive perceptions of concrete models. Pre-service teachers argued that they would use concrete models in their professional lives and that these models were indispensable for mathematics education. In addition, advantages and disadvantages for mathematics educators regarding the use of concrete models are presented.

## Introduction

Educational curricula commonly involve learning scientific models (Justi and Gilbert, 2002; Kim, 2020). These models contribute to the professional development of both researchers and teachers (Harrison, 2001). Since mathematics is the basis of the rapid progress of technology and science, people in our age need to be able to use mathematics in their daily life in order to adapt to these developments. However, although mathematics is an important science for society, many people perceive mathematics as irrelevant to daily life. According to Muller and Burkhardt (2007), many people think of mathematics only as the grammar of the language of mathematics, its rules, and minor operations. Therefore, one of the problems in mathematics education stems from the traditional view that regards the nature of mathematics as a detached, non-everyday pursuit. Since students learn mathematics to be successful in exams, not in daily life, mathematics turns into an unpleasant course.

This purpose of mathematics education has an impact on the structuring of mathematics classes. Therefore, in mathematics teaching, more examples should be considered in which students can understand the importance of mathematics in their daily living and solve real mathematical problems in their daily lives (Kaiser and Schwarz, 2006). Strong, shareable, and reusable concrete models used by mathematicians are the most important cognitive objects of mathematics education (Lesh and Yoon, 2007; Nugroho and Jailani, 2019). Concrete models are objects that concretize abstract mathematical concepts and mathematical tools formed specifically for this purpose such as pictures and objects from real life; they are objects that can be touched and moved (Cramer and Wyberg, 2009).

In teaching practices enriched with concrete models, students’ tendency to see mathematics as a discipline isolated from real life is eliminated, and they are made to realize that a way of thinking that produces solutions to real-life problems through models is a dimension of mathematics (Milli Eğitim Bakanlığı [MEB], 2018). Concrete models are an interdisciplinary issue that covers many other fields besides mathematics (Lingefjärd, 2007) as the source of modeling problems may be different disciplines other than mathematics (Cheng, 2001). Interdisciplinary problem solving, on the other hand, includes the subjects of mathematics, engineering, and science, which enable students to overcome real-life problems that they encounter now or will encounter in the future (Mousoulides and English, 2011).

Effective classroom management skills are essential for pre-service teachers. Unfortunately, many teachers do not receive adequate classroom management training before embarking on their teaching careers and feel unprepared for the demands of managing student behavior in their classrooms. Although mathematics teaching practices enriched with concrete models in effective classroom management have been defined, effective classroom management has an important place between research basis and teacher education requirements. As a result, many prospective teachers need to be prepared to effectively manage student behavior upon completion of a teacher preparation program, as they are not exposed to classroom management content.

In summary, while teaching practices enriched with concrete models represent a process, concrete models are the result of repeated processes in which students express their opinions, interpret, test, and organize (Carreira and Baioa, 2011). From these perspectives, the purpose of teaching practices enriched with concrete models is to enable students to better understand mathematical concepts, to teach them to solve and formulate unique problems, to become aware of their critical and creative aspects, and to contribute to shaping their attitudes toward mathematics (Blum, 2002; Blum et al., 2007). According to Aydın (2008), the purpose of teaching practices enriched with concrete models is to predict, explain, describe, and understand different aspects of the real world.

With the changes in mathematics education in recent years, learning environments in which students represent knowledge with concrete models and the necessity of learning by discovering and understanding are emphasized. Concrete models can be used in mathematics classes to realize this situation and to provide a better understanding of mathematical concepts. In order for concrete models to be effective in teaching mathematics, teachers must have the skills to choose appropriate materials and manipulatives and to use them effectively (Thompson, 1994). Therefore, both teachers and pre-service teachers need to recognize concrete models that can be used in mathematics teaching and to know how to use them in the learning-teaching process (Akkaya et al., 2009).

Thompson (1994) conducted a series of studies on the usage of concrete models in mathematics education and these studies made their way to today since the first publications of Bruner and Dienes. In addition, he reported an important success in teaching adding and subtracting algorithms with elementary school students. To present an effective education in mathematics teaching, manipulatives, concrete models, shapes, computer-assisted education, mathematical games, worksheets, and books are indispensable elements of this process (Drews and Hansen, 2007).

## Pedagogical framework(s)

Concrete models are objects that facilitate the problem-solving skills of students. They are effective in terms of both cost and benefit. Concrete models are concrete objects that describe real-world information. They positively affect the performance of students on math problems. Teaching practices enriched with concrete models in mathematics teaching affect students’ perceptions positively. However, concrete models cannot be used effectively enough due to the intensive school mathematics curriculum and limited time (McNeil et al., 2009).

The use of concrete objects in mathematics teaching offers a new perspective. It enables students to do mathematics without understanding mathematics (Kim, 2020). It may be difficult to express the sharp distinction between concrete and abstract models in mathematics teaching by accepting that concrete models are effective. Instead, manipulatives can be used. Studies on how young children comprehend symbolic relationships are possible with teaching practices enriched with concrete models (Sterner et al., 2020). Concrete models enable students to access concepts and objects that are difficult to attain in mathematics teaching (McNeil et al., 2009).

Cramer and Wyberg (2009) argue that teachers should use multiple models to support subject teaching in mathematics. They also emphasize that concrete models improve students’ calculation skills. For students who have problems manipulating their mental images, concrete models can positively reduce this difficulty. Concrete models prevent students from using wrong strategies and reduce misconceptions. There may be a misuse of teachers at the point of applying concrete models in mathematics teaching. Concrete models may have their strengths and limitations. Using multiple concrete models can be an effective strategy to enable students to learn meaningfully (Salimi et al., 2020).

The Concrete-Representational-Abstract (CRA) approach is a three-stage strategy that promotes overall conceptual understanding using sensory teaching techniques that introduce new concepts. Each stage is based on previously taught concepts. This approach is an effective one used in mathematics courses and for teaching in many different areas of mathematics, which includes an exploration-learning strategy that helps students transition between conceptual and content knowledge. With this approach, concrete models gain importance and make positive contributions to students’ learning in the transition from mathematics to abstract (National Council of Teachers of Mathematics [NCTM], 2020).

Teaching practices enriched with concrete models is a process that offers students the opportunity to communicate with mathematical ideas and develop their teamwork skills. In addition, this process helps teachers to recognize the mathematical thinking, skills, and abilities of a wide range of students. Thus, teachers assume the role of facilitator in activities where concrete models are used (Fox, 2006).

Lesh and Doerr (2003) presented their views on the reasons for developing teaching practices enriched with concrete models. Teachers believe that many of the skills they try to give cannot be evaluated with standard tests, and they believe that most of their students are more successful than implied by standard test results with teaching practices enriched with concrete models. In the context of positive psychology, intellectual foundations in the field of educational administration are questioned. In this study, it was aimed to determine the opinions of pre-service mathematics and pre-service primary school teachers regarding mathematics teaching practices enriched with concrete models. Since visual objects are more memorable, it is important that such a study is conducted in order for students to learn abstract mathematics subjects better. In addition, since pre-service teachers will graduate after a short time and will be engaged in teaching activities, they will be able to better transfer these subjects to students by using teaching practices enriched with concrete models.

Teachers’ perceptions of positive psychology are based on the characteristics of positive people, which is a neglected subject of psychology in recent years. An analysis has been made about how positive psychology can be benefited from in the field of humanistic psychology, which is fed by positive psychology, and educational administration (Aydın et al., 2013). While teachers use concrete models in teaching mathematics, their personal, and general efficacy beliefs are directly proportional to their positive psychology perceptions (Kelekçi and Yılmaz, 2015). In addition, since concrete models that provide permanent learning in students also increase problem solving skills, they add a positive meaning and predict positive psychology perceptions of both teachers and students (Anık and Tösten, 2019).

## Materials and methods

### Research design

In the research, the opinions and evaluations of pre-service mathematics and pre-service primary school teachers about the concrete models they designed were examined. The research was designed with a mixed-method in which qualitative and quantitative approaches were used together. By placing Mixed Method Researches in an analysis of some common research paradigms, it is important to complement and meet the increasingly complex needs of contemporary researchers. There are several benefits to adopting Mixed Method Research as it integrates post-positivism and interpretive frameworks by explaining the key principles and key theoretical principles of the mixed methods approach, which includes collecting both quantitative and qualitative data in response to research questions (Saraswati et al., 2021). This integrated method includes collecting, analyzing, and combining qualitative and quantitative data in one or more stages (Hesse-Biber, 2010). Fully mixed concurrent dominant status design: F2 is used in our study. A fully mixed concurrent dominant status design involves conducting a study that mixes qualitative and quantitative research within one or more of, or across the aforementioned three components in a single research study. In this design, the quantitative and qualitative phases are mixed concurrently at one or more stages or across the stages (Leech and Onwuegbuzie, 2009).

### Participants

Regarding the characteristics of the mixed research study, it was tried to reach the widest possible participant group in the participatory quantitative research section. During the scale development phase of the research, 441 pre-service teachers studying mathematics and pre-service primary school teaching departments of the education faculties of universities in Turkey and Cyprus were surveyed. Of the pre-service teachers participating in the research, 54% are female and 46% are male. In the qualitative part of the research, in order to conduct in-depth research, it was aimed to select information-rich situations in the context of the purpose of the study, and 44 pre-service teachers were reached by using the purposive sampling method. In the qualitative aspect of the research, it was aimed to reveal the opinions of pre-service teachers on “Mathematics Teaching Practices Enriched with Concrete Models.” For this, the interview technique, one of the qualitative research designs, was used. The data obtained from the interview at the end of the qualitative research were analyzed by using the content analysis technique. Qualitative research is frequently preferred in accordance with research subjects in terms of making events and perceptions in natural environments, being holistic, and providing in-depth information about the subject (Yıldırım and Şimşek, 2013). In the present study, the “Mathematics Teaching Practices Enriched with Concrete Models” course was given 3 h per week for 8 weeks (3 × 8 = 24 h) to pre-service mathematics and pre-service primary school teachers by using the Moodle system with the distance education method. In the research, the distance education method was preferred to perform the teaching due to the COVID-19 pandemic and to reach more pre-service teachers.

### Data collection tools

In the study, the “Mathematics Teaching Practices Enriched with Concrete Models Perception Scale” was used as a data collection tool. While preparing the questionnaire, the literature was surveyed and the form consisting of 26 items and 4 open-ended questions was prepared by applying the opinions of three field experts and a qualitative research expert. In order to apply the questionnaire, the necessary permissions were obtained *via* e-mail, and after the permissions were obtained, the questionnaires were implemented with the help of Google Forms. The questionnaire form consists of 26 items and is in a 5-point Likert type. For each of the 26 items, one is asked to choose one of the options of strongly disagree, disagree, neither disagree nor agree, agree, and strongly agree. The ninth question was prepared as negative. While calculating the scale score, the answers were given (5-point Likert type) 1, 2, 3, 4, and 5 points, respectively. Reliability test was applied to the scale in the study and the Cronbach’s alpha value was found as 0.81. It is said that this reliability value for the scales is good (Büyüköztürk et al., 2017).

In this research, it is aimed to evaluate pre-service mathematics teachers’ and pre-service primary school teachers’ opinions and perspectives on mathematics teaching practices enriched with concrete models. In this direction, answers to the following open-ended questions were sought:

1. Has the concrete model you developed contributed to your professional development? Please explain.
2. Are concrete models suitable for every subject in mathematics teaching? Why? Why not?
3. What are the advantages and disadvantages of concrete models in teaching mathematics?
4. Do you intend to use concrete models in your professional life? Why? Why not?

The analysis results obtained in the research were tested by coding with the consensus of two experts with qualitative research experience. The researcher first coded the interview records of the research. These codings were repeated by two independent experts apart from the researchers. The reliability formula of Miles and Huberman (1994) was used to calculate the reliability of the interviews.

As a result of the coding, the reliability level of the “Interview Questions for Pre-service Teachers” was found to be 84% with the first expert and 88% with the second expert. According to Yıldırım and Şimşek (2013), a degree of reliability of 70% is sufficient. Since this rate was exceeded in this study, it was concluded that the data obtained after the qualitative research were reliable.

### Learning environment

During the 8-week education period, the students were taught about concrete models; on the condition, that one topic was covered each week. These topics are respectively: Explanation and Transformation of Concrete Models, Interpreting Concrete Models, Developing and Solving Concrete Models, Verifying Concrete Models, Using Concrete Models, The Importance of Concrete Models in Mathematics Curriculum, The Place of Concrete Models in the Mathematics Curriculum, and Construction and Presentation of Concrete Models. After the 8-week course, each of the 44 pre-service mathematics and pre-service primary school teachers was asked to develop a concrete model as a project on a topic they chose. Thus, while the pre-service teachers were given more information about the research topic, they were provided answers to the scale and qualitative questions these were more realistic.

**FIGURE 1:**
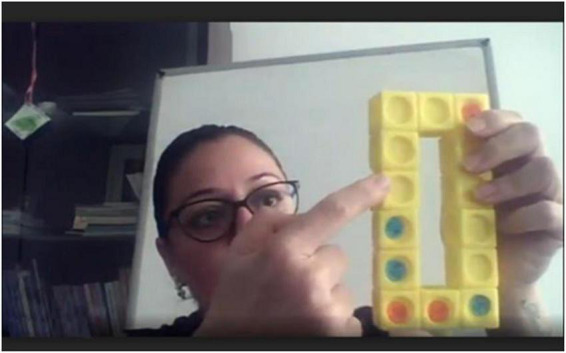
Sample lectures made with concrete models (by MC).

**FIGURE 2:**
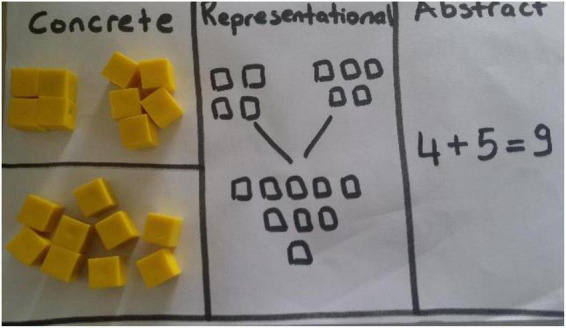
The transition from concrete to abstract and its representation (by MC).

In Figure 1, the training made on the Moodle platform is given. In the training, it was tried to comply with the standards specified by National Council of Teachers of Mathematics (NCTM). In Figure 2, the projects produced by the pre-service teachers at the end of the lesson are given.

Due to the COVID-19 pandemic, teacher candidates were asked to send the concrete models they produced to the authors’ address by courier.

### Data analysis

“Mathematics Teaching Practices Enriched with Concrete Models Perception Scale” was applied after 8 weeks of training to collect the necessary data for the statistical analysis of the research problem. The collected data were transferred to a computer environment and analyzed with the SPSS 24.0 program. The mean and standard deviation values of each item were calculated. By collecting quantitative data with the related scale, the opinions of pre-service mathematics teachers and pre-service primary school teachers about concrete models they developed themselves were revealed. After the qualitative data on open-ended questions were analyzed with frequency values, the obtained findings guided the collection of quantitative data. Thus, exploratory design, one of the mixed methods, was used in the research. The Kaiser-Meyer Olkin (KMO) test used to explain whether the selected sample is sufficient takes a value between zero and one. KMO value must be greater than 0.50. With a value of 0.905, the sample is considered well suited for factor analysis. If *p* < 0.05 as a result of Bartlett’s test, it is seen that the result is significant (Büyüköztürk et al., 2017).

Before the scale was applied to the pre-service teachers, a pre-application was made with the questionnaire form. After the validity and reliability analyzes were made, it was applied to the participants again to obtain descriptive findings. In addition, exploratory factor analysis was performed while developing the perception scale. Factor loads of “Mathematics Teaching Practices Enriched with Concrete Models Perception Scale” are given in Table 1.

**TABLE 1:** Factor loads of mathematics teaching practices enriched with concrete models perception scale.

Factor loads					
**Item**	**1st factor**	**2nd factor**	**3rd factor**	**4th factor**	**5th factor**
Item 1	0.825				
Item 2	0.816				
Item 3	0.799				
Item 4	0.796				
Item 5	0.787				
Item 6	0.777				
Item 7	0.738				
Item 8	0.690				
Item 9	0.627				
Item 10		0.803			
Item 11		0.791			
Item 12		0.773			
Item 13		0.734			
Item 14		0.728			
Item 15		0.663			
Item 16		0.603			
Item 17			0.825		
Item 18			0.788		
Item 19			0.749		
Item 20			0.689		
Item 21			0.593		
Item 22				0.766	
Item 23				0.664	
Item 24				0.592	
Item 25					0.834
Item 26					0.753

**FIGURE 3:**
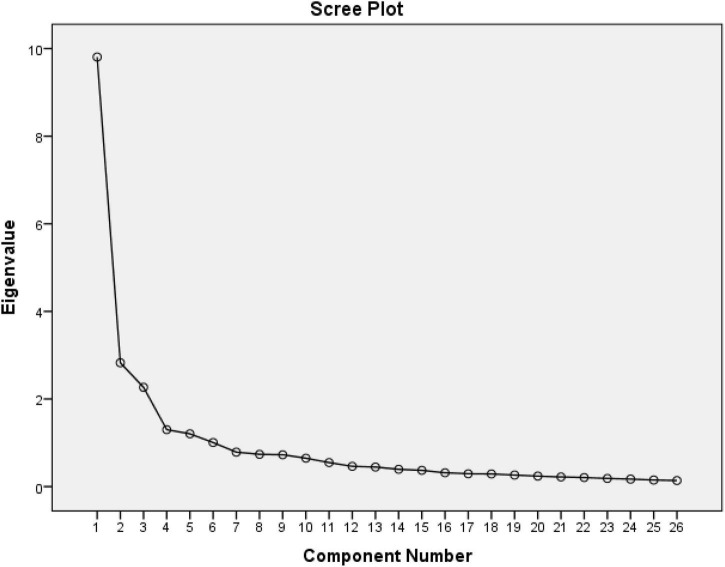
Scree plot graphic.

As a result of factor analysis and examination of the breakpoints in the Scree Plot Graphic (Figure 3), it was understood that the scale was divided into five sub-factors. However, after Varimax rotation in the factor analysis, the total variance explained by the five factors is 66.940%, which is good (Büyüköztürk et al., 2017).

Considering the factors in the Mathematics Teaching Practices Enriched with Concrete Models Perception Scale, which emerged in the distribution of 26 items, the following can be said:

The first factor is the “benefit” factor, the second factor is the “usage” factor, the third factor is the “planning” factor, the fourth factor is the “sustainability factor,” and the 5th factor is the curriculum factor. These factors include items related to the evaluation of mathematics teaching practices enriched with concrete models.

## Results

The findings obtained from the analysis of the data were presented in tables and comments were made according to these tables. The descriptive statistical findings of the Mathematics Teaching Practices Enriched with Concrete Models Perception Scale applied to “Pre-service mathematics and pre-service primary school teachers” and the answers to the open-ended questions were given in tables by conducting content analysis.

In Table 2i, it is seen that the perceptions of most of the pre-service teachers about concrete models are in the form of “Agree” ($\bar{X}=3.41;Sd=0.265)$. In general, pre-service teachers responded positively to the items in the benefit sub-factor and said they agree ($\bar{X}=3.67;Sd=0.415)$. They also stated that concrete models (CM) helps to understand mathematical concepts more clearly, makes it easier for students to learn knowledge, provides permanent learning, enables meaningful learning, increases students’ interest in the class, creates a fun learning environment, and develops students’ psychomotor skills. However, on the contrary, pre-service teachers stated that designing CM would take time instead of saving time, and they said that CM developed the imagination of the students.

**TABLE 2i:** Mathematics teaching practices enriched with concrete models perception scale.

	Items	$\bar{X}$	SD
	**Benefit**	3.67	0.415
1	CM helps to understand mathematical concepts more clearly.	3.78	0.935
2	CM makes it easier for students to learn knowledge.	3.65	0.874
3	CM provides permanent learning.	3.79	0.918
4	CM enables meaningful learning.	3.41	0.991
5	CM saves time.	2.45	0.981
6	CM increases students’ interest in the class.	3.95	0.832
7	CM creates a fun learning environment.	3.91	0.880
8	CM develops students’ psychomotor skills.	4.12	0.88
9	CM does not develop students’ imaginations.	1.95	0.808
	**Usage**	3.31	0.376
10	In order for CM to be used effectively. Pre-service teachers must be equipped.	3.17	1.055
11	CM can be moved.	2.89	0.705
12	CM can be used during an activity in the course.	3.37	1.268
13	CM can be used to increase visibility.	3.88	0.879
14	CM can be used to attract attention at the beginning of the class.	3.70	0.807
15	CM can be used for reinforcement at the end of the class.	3.18	0.74
16	CM can be used for assessment at the end of the class.	2.97	0.742
	**Planning**	2.75	0.519
17	CM is easy to prepare.	2.24	0.842
18	Concrete models are durable.	2.23	0.814
19	Concrete models are compatible with STEM.	3.05	0.721
20	CM is visually aesthetic.	3.18	0.804
21	CM can be prepared with easily available materials.	3.03	1.185
	**Sustainability**	3.67	0.563
22	CM contains real-life objects.	3.54	0.85
23	Concrete models can be improved by making some changes.	3.84	0.974
24	CM can be used in other subjects by making some changes on it.	3.63	0.764
	**Curriculum**	3.72	0.718
25	CM are objects that concretize abstract mathematical concepts.	3.74	1.005
26	It is necessary to use concrete models in mathematics teaching.	3.69	0.851
	Total	**3.41**	**0.265**

Pre-service teachers said neither agree nor agree on the items in the usage sub-factor ($\bar{X}=3.31;Sd=0.376)$. In addition, pre-service teachers were undecided about the “In order for CM to be used effectively, Pre-service teachers must be equipped,” “CM can be moved,” “CM can be used during an activity in the course,” “CM can be used for reinforcement at the end of the class,” and “CM can be used for assessment at the end of the class” items. However, in this sub-factor, it was emphasized that CM could be used to increase visibility and attract attention at the beginning of the lesson.

Pre-service teachers said neither agree nor agree on the items in the planning sub-factor ($\bar{X}=2.75;Sd=0.519)$. In addition, pre-service teachers were undecided about the “CM is easy to prepare,” “Concrete models are durable,” “Concrete models are compatible with Science, Technology, Engineering, and Mathematics (STEM),” “CM is visually aesthetic,” and “CM can be prepared with easily available materials” items.

In general, pre-service teachers said an “agree” response to the items in the sustainability sub-factor ($\bar{X}=3.67;Sd=0.563)$. In addition, pre-service teachers were agree with the “CM contains real-life objects,” “Concrete models can be improved by making some changes,” “CM can be used in other subjects by making some changes on it” items.

In addition, pre-service teachers said an “agree” response to the items in the curriculum sub-factor. In addition, pre-service teachers were agree with the “CM are objects that concretize abstract mathematical concept” and “It is necessary to use concrete models in teaching” items.

Below are the findings regarding the open-ended questions in Table 2ii. After the qualitative research, the results obtained depending on the coding are given in the form of themes and sub-themes. Contribution, causes, suitability, advantages, disadvantages, and effect on learning were determined as the main themes. In Table 2ii, one can be seen that approximately 97.8% of the pre-service teachers answered “yes” to the first question and that mathematics teaching practices enriched with concrete models contributed to their professional development.

**TABLE 2ii:** Pre-service teachers’ opinions on questions.

Question 1(a)		
Contribution	*f*	%
Yes	43	97.8
No	1	2.2
Total	**44**	**100**

**Question 1(b)**		

Causes	f	%
Concretizing the topic covered	10	22.73
Providing richness to the topics to be covered	8	18.18
Creating an effective learning environment	7	15.91
No answer	5	11.36
Being able to motivate students	4	9.09
Being able to deliver an effective and lasting class	4	9.09
Providing productivity to professional development	3	6.82
Providing learning by doing	3	6.82
Total	**44**	**100**

**Question 2**		

Suitability	f	%
Yes	38	86.36
It is possible to design a concrete model for every subject	28	63.64
Using creativity/Concrete models can be designed for any subject	6	13.64
No comment	4	9.09
No	6	13.64
More suitable for primary school students	3	6.82
It may not be suitable for every subject	2	4.55
In some subjects, the presentation method alone is sufficient	1	2.27
Total	**44**	**100**

**Question 3(a)**		

Advantages	f	%
Concretizing abstract knowledge	30	68.18
Providing permanent learning	15	34.09
Enabling students to develop positive attitudes toward mathematics	9	20.45
Increasing student motivation	8	18.18
Creating a learning environment by doing and living	4	9.09
Being fun	4	9.09

**Question 3(b)**		

Disadvantages	f	%
It requires financial means	19	43.18
It should be used under teacher control	10	22.73
It can cause time loss in crowded classrooms	9	20.45
There is no concrete model suitable for every subject	6	13.64
Total	**44**	**100**

**Question 4**		

Effect on learning	f	%
To ensure permanent learning	10	22.73
To increase motivation	7	15.91
To make the class productive	6	13.64
To prevent abstract knowledge from being memorized	6	13.64
To make the class fun	5	11.36
To enable critical thinking	4	9.09
To enable creative thinking	3	6.82
To create an environment for learning by doing-living	3	6.82
Total	44	100

In the explanation part of the first question in Table 2ii above, the direction of the contribution of mathematics education applications enriched with concrete models to the professional development of pre-service teachers is described. In terms of frequency value, the first rank is occupied by making the topic covered concrete whereas the last rank is occupied by contributing in terms of ensuring learning by doing-living.

According to Table 2ii, it was seen that 86% of the pre-service teachers gave “yes” and 14% gave “no” answers to the question “whether concrete models are suitable for all subjects” along with their reasons.

The statements in Table 2ii indicate the advantages of teaching practices enriched with concrete models applied by pre-service teachers. The resulting sub-themes are “Concretizing abstract knowledge,” “Providing permanent learning,” “Enabling students to develop positive attitudes toward mathematics,” “Increasing student motivation,” “Creating a learning environment by doing and living,” and “Being fun.”

The statements in Table 2ii are among the disadvantages of teaching practices enriched with concrete models applied by pre-service teachers. The resulting sub-themes are “It requires financial means,” “It should be used under teacher control,” “It can cause time loss in crowded classrooms,” and “There is no concrete model suitable for every subject.”

All of the pre-service teachers stated that they intended to use concrete models in their professional lives. Table 2ii above shows the opinions of pre-service teachers about why they wanted to use mathematics teaching applications enriched with concrete models in their professional lives. The first among these is the statement “To ensure permanent learning.” In addition, they plan to use it in their professional lives to increase student motivation. In addition, pre-service teachers think that CM makes the lesson productive, prevents memorization of abstract information, makes the lesson fun, provides critical-creative thinking, and provides learning by doing-living.

## Discussion, conclusion, and recommendations

In this part of the research, the conclusion drawn from the findings obtained as a result of the study and suggestions in line with these conclusion are provided.

### Discussion and conclusion

It is seen that the opinions of the majority of pre-service mathematics teachers and pre-service primary school teachers about mathematics teaching practices enriched with concrete models are positive in the form of “Agree.” In addition, it was observed that pre-service teachers did not use negative expressions in items such as concrete models containing real-life objects. On the contrary, it has been determined that these models are objects that embody abstract mathematical concepts, help students understand mathematical concepts more clearly, and enable students to learn more information. Pre-service teachers also emphasized that concrete models provide easy, permanent, and meaningful learning, increase students’ interest in the lesson and that they should be used in mathematics teaching. At the same time, they said that these models create a fun learning environment, increase the psychomotor skills of the students and that these models are aesthetic.

About 97.8% of the pre-service teachers answered “yes” to the first open-ended question. Accordingly, it was seen that mathematics teaching practices enriched with concrete models contribute to their professional development. This result is in parallel with the study of Thompson (1994). In the explanations part, as regards the contributions of mathematics education applications enriched with concrete models to their professional developments, pre-service teachers mentioned “concretizing abstract knowledge” with the highest frequency and “ensuring learning by doing-living” with the lowest frequency.

Almost all of the pre-service teachers answered “yes” to the question “Are concrete models suitable for every subject in mathematics teaching?” Among the advantages of mathematics teaching practices enriched with concrete models pointed out by pre-service teachers, in line with Nugroho and Jailani (2019), it is mentioned that it concretizes abstract knowledge and provides permanent learning. The fact that students develop a positive attitude toward mathematics is in parallel with Blum (2002) and Blum et al. (2007). They also provided statements such as increasing motivation, creating a learning environment by doing and living, and being fun. Larson (1983) underlines that no conflict really exists between covering the usual content of a course in mathematic methods and at the same time encouraging positive attitudes toward mathematics and combating math anxiety.

Among the disadvantages of mathematics teaching practices enriched with concrete models applied by pre-service teachers, the following were pointed out: it requires financial means, it should be used under teacher control, it can lead to time loss in crowded classrooms, and there is no concrete model suitable for every subject. This result shows that we reached the same conclusion as McNeil et al. (2009).

All of the pre-service teachers stated that they intended to use concrete models in their professional lives. This result proves that concrete models are an indispensable part of mathematics education, as indicated in the study of Drews and Hansen (2007) and Kim (2020). In the pre-service teachers’ opinions on why they wanted to use mathematics teaching practices enriched with concrete models in their professional lives, the expression “to provide permanent learning” takes the first place. It is followed by the desire to increase student motivation, make the class productive, and prevent abstract knowledge from being memorized. This result is in parallel with Nugroho and Jailani (2019). In addition, as in the study of Cramer and Wyberg (2009), they intend to use it in their professional lives to make the class more fun, to provide critical-creative thinking, and to ensure learning by doing-living. It is also known that positive psychology affects school-age children positively, both their attitudes and motivations (Akdoğan, 2021). It can be thought that the use of concrete models in mathematics teaching creates a positive psychology context for children’s motivation and attitudes.

### Recommendations

• It can be argued that the lack of a concrete model suitable for every subject can be compensated for with Computer-Aided Education called “semi-concrete.”
• The fact that there are very few works in the literature on mathematics teaching practices enriched with concrete models shows that more studies should be done on this subject.
• The fact that all teachers will use Mathematics Teaching Practices Enriched with Concrete Models in their professional lives shows that, depending on international education policies, teachers should take new learning approaches practically in undergraduate courses in education faculties and that this is inevitable in mathematics education.
• Senior students studying in education faculties of universities can be given such models as homework and projects for research purposes, and they can be asked to prepare and present class examples suitable for these models. However, knowledgeable people must evaluate these sample lessons. These sample lessons need to be evaluated by knowledgeable people. Mathematics is said to be one of the most difficult subject in all countries of the world. Especially since the mathematics curriculum is intense and time is limited, mathematics and primary school teachers need to make choices that will use time economically while determining an effective teaching model. They can apply mathematics teaching practices enriched with concrete models as a method that will provide important results in students’ academic success and save time.
• Using mathematics teaching practices enriched with concrete models for using problem-solving strategies, making effort to solve problems, loving problem solving, and having self-confidence in problem-solving to improve students’ problem-solving skills and structure in mathematics and especially geometry classes can play a key role in eliminating low student performance.

## Data availability statement

The original contributions presented in this study are included in the article/supplementary material, further inquiries can be directed to the corresponding author.

## Ethics statement

The studies involving human participants were reviewed and approved by Ethical Committee Board of Near East University. The patients/participants provided their written informed consent to participate in this study. Written informed consent was obtained from the individual(s) for the publication of any potentially identifiable images or data included in this article.

## Author contributions

All authors listed have made a substantial, direct, and intellectual contribution to the work, and approved it for publication.
